# Serum D-dimer, albumin and systemic inflammatory response markers in ovarian clear cell carcinoma and their prognostic implications

**DOI:** 10.1186/s13048-020-00693-w

**Published:** 2020-08-08

**Authors:** Wei Chen, Siyuan Zhong, Boer Shan, Shuling Zhou, Xiaohua Wu, Huijuan Yang, Shuang Ye

**Affiliations:** 1 Department of Gynecologic Oncology, Fudan University Shanghai Cancer Center, Shanghai, China; 2 Department of Obstetrics and Gynecology, Minhang Hospital, Fudan University, the Central Hospital of Minhang District, Shanghai, China; 3 Department of Pathology, Fudan University Shanghai Cancer Center, Shanghai, China; 4 Department of Oncology, Shanghai Medical College, Fudan University, Shanghai, 200032 China

**Keywords:** Ovarian neoplasms, Clear cell carcinoma, D-dimer, Albumin, Neutrophil to lymphocyte ratio, Platinum resistance, Recurrence, Survival

## Abstract

**Background:**

This study attempts to evaluate whether preoperative systemic inflammatory response (SIR) markers or other hematological variables, such as albumin, D-dimer, and carbohydrate antigen 125, play roles in predicting chemotherapy response and survival outcome in patients with ovarian clear cell carcinoma (OCCC).

**Methods:**

Preoperative leukocyte differential counts, as well as platelet, serum albumin, plasma D-dimer and CA-125 levels, were measured in patients with FIGO IC-IV ovarian clear cell cancer. The correlations of these hematological biomarkers with clinicopathological features, chemotherapy response, and survival outcomes were further analyzed. Survival time was estimated using the Kaplan-Meier model, whereas Cox regression was conducted for multivariate analysis.

**Results:**

Among the 84 patients, 28.6% were classified as platinum resistant, and 69.0% were platinum sensitive. Preoperative CA125, albumin, and D-dimer levels; neutrophil to lymphocyte ratios (NLR); and monocyte to lymphocyte ratios were significantly correlated with FIGO stage, residual tumor, and platinum response. Platelet to lymphocyte ratio was not related to platinum response (*P* = 0.060). The median follow-up time was 28 months (range, 1 to 128 months). Preoperative CA125, albumin, and D-dimer levels were significant prognostic factors for overall survival (OS) and progression-free survival (PFS). In the univariate analysis, only NLR exhibited prognostic significance for PFS (*P* = 0.007). Multivariate analysis indicated that D-dimer > 3.27 (*P* = 0.001 for OS; *P* = 0.040 for PFS) and albumin < 39.6 (*P* = 0.005 for OS and *P* = 0.041 for PFS) retained significance.

**Conclusions:**

Preoperative NLR has some predictive value for platinum resistance in patients with IC-IV stage OCCC but has little predictive effect on prognosis. Elevated D-dimer and reduced albumin might be potential biomarkers for worse response to first-line platinum-based chemotherapy and poor clinical outcomes.

## Background

Epithelial ovarian cancer (EOC) is the eighth most common cause of female cancer death worldwide [1]. Ovarian clear cell carcinoma (OCCC) is a distinct histological subtype that accounts for 5–25% of all EOC and is more commonly observed in Asian women [2, 3]. Although the prognosis for patients with stage I OCCC is relatively good, patients with stage IC-IV OCCC presents much poorer prognoses than patients with serous carcinoma due to its disease aggressiveness and chemotherapy resistance [3, 4]. The factors, such as the International Federation of Gynecology and Obstetrics (FIGO) stage, residual tumor, and platinum response, influence treatment outcomes in OCCC [5]. However, these factors are limited to be confirmed after surgery or chemotherapy. The current standard treatment for EOC remains surgery and platinum-based cytotoxic chemotherapy. Generally, OCCC patients receive routine treatment, while platinum-resistant patients derive minimal benefit from it but increased morbidity and costs. Clinically useful preoperative prognostic factors for early identification of chemotherapeutic responses are needed to improve clinical outcomes and decrease toxicity in stage IC-IV OCCC.

Albumin, D-dimer, and systemic inflammatory response (SIR) markers, such as neutrophil-to-lymphocyte ratio (NLR), monocyte-to-lymphocyte ratio (MLR) and platelets-to-lymphocyte ratio (PLR), are easily accessible and inexpensive to evaluate before initial treatment. Albumin is a significant prognostic factor for overall survival of ovarian cancer [6]. Plasma D-dimer levels, which are significantly elevated in patients with OCCC, are associated with the incidence of deep venous thrombosis [7] as well as clinical progression and poor prognosis in malignancies, including ovarian cancer [8, 9]. Numerous studies have shown that elevated NLR is linked to poor prognosis among patients with solid tumors, including gynecologic cancers [10, 11], and markers of systemic inflammatory response could provide useful prognostic information of overall survival in patients with OCCC [12, 13].

However, evidence for the use of available biomarkers preoperatively as predictors of outcome in patients with OCCC receiving chemotherapy is lacking. The purpose of the current study was to determine whether preoperative hematological biomarkers, such as albumin, D-dimer, and carbohydrate antigen 125 (CA125), or SIR markers could play a role in predicting response to chemotherapy and survival outcome.

## Materials and methods

### Patients

The institutional review board approved the study, and the requirement for written informed consent was waived due to its retrospective design. We searched the Electronic Medical Record system to include all the patients who received initial surgery and were diagnosed with OCCC at our institution from 2008 to 2018. The inclusion criteria were listed as follows: 1) A pathologically confirmed diagnosis of OCCC; 2) No preoperative treatment, including chemotherapy; 3) Without autoimmune diseases, systemic diseases, and serious diseases; 4) Without any sign of infection.

Early-stage patients received comprehensive staging surgery, while advanced patients underwent debulking surgery. All patients received paclitaxel and carboplatin-based chemotherapy. Platinum resistance was identified as progression within 6 months after the last platinum treatment, whereas platinum-refractory status was defined as progression during chemotherapy. Overall survival (OS) was calculated as the time interval from initial surgery to death or last contact. Progression-free survival (PFS) was defined as the time interval from initial surgery to the date of the first recurrence.

The time interval from blood collection to surgery is typically less than 7 days. Preoperative leukocyte differential counts (neutrophils, monocyte, and lymphocyte), platelet, serum albumin, plasma D-dimer and CA-125 were retrospectively abstracted from the medical records. NLR was defined as absolute neutrophil count divided by absolute lymphocyte count. MLR defined as was the ratio of absolute monocyte count and absolute lymphocyte count, and PLR was defined as absolute platelet count divided by absolute lymphocyte count.

### Statistical analysis

Descriptive statistics were used to present clinicopathological variables. Medians and ranges are reported for continuous variables, while proportions are used for categorical data. Receiver operating characteristic (ROC) curves were used to obtain optimal albumin, D-dimer, CA125 and SIR marker cutoff values for predicting platinum response. Baseline characteristics were compared using Mann-Whitney U test for skewed data. Survival time was estimated using the Kaplan-Meier model, whereas Cox regression was conducted for multivariate analysis. Variables with statistical significance of univariate analysis were included in multivariate analyses. All *P*-values reported were two-tailed, and *P* < 0.05 was considered statistically significant.

ROC curves and Kaplan-Meier curves were plotted using GraphPad Prism (Version 6.0, GraphPad Software, Inc., La Jolla, CA, USA). All other statistical analyses were performed with Statistical Package for Social Science (SPSS) (Version 20.0, SPSS, Inc., Chicago, IL, USA).

## Result

### Relations between preoperative hematological biomarkers and clinicopathological characteristics

A total of 91 OCCC participants who received initial surgery in our institution were identified for inclusion, and seven were excluded based on FIGO IA-IB stage. Eighty-four cases were enrolled in the analysis. The median age of the patients was 52 years (range, 26 to 83 years). In total, 44.0% (37/84) of the patients presented with late-stage tumors (FIGO III + IV). Optimal debulking was achieved in 91.7% (77/84) patients. Eighty-two cases were available for platinum response assessment, and 2 patients were lost to follow-up during chemotherapy. In terms of chemotherapy response, 29.3% (24/82) patients were classified as platinum resistant, and 70.7% (58/82) were classified as platinum sensitive. The median follow-up time was 28 months (range, 1 to 128 months).

Table 1 shows the median and range for leukocyte differential counts, CA125, albumin, D-dimer, and SIR markers based on tumor characteristics. Generally, preoperative CA125, albumin, D-dimer, and SIR markers were significantly associated with FIGO stage, residual tumor, and platinum response. Neutrophilia; monocytosis; lymphopenia; elevated NLR, MLR, PLR, and CA-125 levels; and decreased albumin levels were associated with advanced-stage disease and suboptimal debulking. Preoperative D-dimer levels were not directly linked to the availability of optimal debulking. Elevated CA125, D-dimer, NLR, and MLR levels and low albumin levels were associated with platinum resistance (*P* < 0.05). Interestingly, platelet count was independent of FIGO stage, residual tumor, and platinum resistance. Lymphocyte count was also independent of platinum resistance. Therefore, PLR exhibited no significant difference in platinum-sensitive and platinum-resistance patients (*P* = 0.060).

**Table 1:** Patient characteristics in relation to preoperative blood parameters and SIR marks

Characteristic	N^a^ (%)	CA125^b^ (U/ml)	Albumin ^b^ (g/l)	Leukocyte differential counts ^b^ (k/μl)			Platelet ^b^ (k/μl)	D-dimer ^b^ (mg/l)	Ratio ^b^		
				Lymphocyte	Monocyte	Neutrophil			NLR	MLR	PLR
All cases	84	187.9 (6.5–5000*)	40.7 (25.9–52.1)	1.5 (0.4–2.9)	0.4 (0.03–1.6)	4.1 (1.9–15.2)	299 (124–608)	1.66 (0.19–55.20)	2.7 (1.1–20.3)	0.28 (0.03–2.00)	193.9 (77.5–955.0)
Age (years)											
≤ 52	42 (50)	188.0 (6.5–2854)	40.7 (33.1–49.4)	1.5 (0.6–2.9)	0.4 (0.2–1.0)	4.0 (1.9–12.2)	301 (124–500)	1.66 (0.19–55.20)	2.6 (1.1–20.3)	0.25 (0.12–0.83)	201.7 (77.5–615.0)
> 52	42 (50)	165.0 (11.1–5000*)	40.4 (25.9–52.1)	1.5 (0.4–2.4)	0.4 (0.03–1.6)	4.4 (1.9–15.2)	296 (147–608)	1.66 (0.20–20.00)	2.8 (1.1–20.3)	0.32 (0.03–2.00)	191.0 (92.1–955.0)
*P* value		0.589	0.056	0.874	**0.039**	0.826	0.871	0.756	0.826	0.053	0.940
FIGO stage											
IC-II	47 (56.0)	70.5 (6.5–1930.0)	43.6 (29.6–52.1)	1.5 (0.9–2.9)	0.4 (0.03–1.0)	3.6 (1.9–10.7)	292 (124–500)	1.49 (0.19–55.20)	2.2 (1.1–7.1)	0.21 (0.03–0.67)	167.5 (77.5–500.0)
III-IV	37 (44.0)	276.9 (45.6–5000*)	39.2 (25.9–49.2)	1.4 (0.4–2.4)	0.4 (0.3–1.6)	4.6 (1.9–15.2)	311 (147–608)	2.61 (0.69–11.03)	3.2 (1.1–20.3)	0.33 (0.18–2.00)	230.0 (101.7–955.0)
*P* value		**< 0.001**	**< 0.001**	**0.031**	**0.002**	**0.009**	0.320	**0.001**	**0.001**	**< 0.001**	**0.006**
Residual tumor (cm)											
0	63 (75.0)	157.4 (6.5–5000*)	41.7 (28.3–52.1)	1.5 (0.6–2.9)	0.4 (0.03–1.6)	3.8 (1.9–15.2)	292 (124–608)	1.66 (0.19–55.20)	2.4 (1.1–7.1)	0.25 (0.03–0.70)	173.5 (77.5–500)
≤ 1	14 (16.7)	371.4 (45.6–1845.3)	38.4 (29.0–43.7)	1.4 (0.9–2.4)	0.5 (0.3–0.9)	4.7 (2.7–6.6)	318 (202–593)	2.53 (0.69–9.30)	3.2 (1.3–5.7)	0.35 (0.19–0.82)	219.8 (107.1–539.1)
> 1	7 (8.3)	475.1 (151.6–1866.0)	33.2 (25.9–49.2)	0.9 (0.4–1.8)	0.6 (0.3–1.4)	8.1 (2.7–12.2)	342 (253–382)	3.29 (0.75–6.06)	8.2 (2.8–20.3)	0.67 (0.39–2.00)	383.3 (190.0–955.0)
*P* value		**0.007**	**0.006**	**0.008**	**0.002**	**0.005**	0.314	0.064	**< 0.001**	**< 0.001**	**0.004**
Platinum response											
Sensitive	58 (69.0)	124.6 (6.47–5000*)	41.7 (29.0–52.1)	1.5 (0.6–2.9)	0.4 (0.03–1.0)	3.8 (1.9–10.7)	279 (124–500)	1.66 (0.19–20.00)	2.4 (1.1–7.1)	0.25 (0.03–0.70)	176.7 (77.5–500.0)
resistant	24 (28.6)	294.3 (38.21–1866.0)	39.2 (25.9–49.2)	1.5 (0.4–2.4)	0.5 (0.2–1.6)	4.9 (2.1–15.2)	316 (202–608)	3.82 (0.44–55.20)	3.2 (1.1–20.3)	0.35 (0.15–2.00)	214.3 (126.3–955.0)
*P* value		**0.002**	**0.012**	0.400	**0.011**	**0.003**	0.157	**< 0.001**	**0.002**	**0.004**	0.060

*Abbreviations: SIR* systemic inflammatory response, *NLR* neutrophil to lymphocyte ratio, *MLR* monocyte to lymphocyte ratio, *PLR* platelet to lymphocyte ratio

*P* values with statistical significance were denoted

^a^ Categorical data are shown in absolute value and proportion

^b^ Continuous variables are represented by median and range

* The upper limit of CA125 detection is 5000

**Fig. 1:**
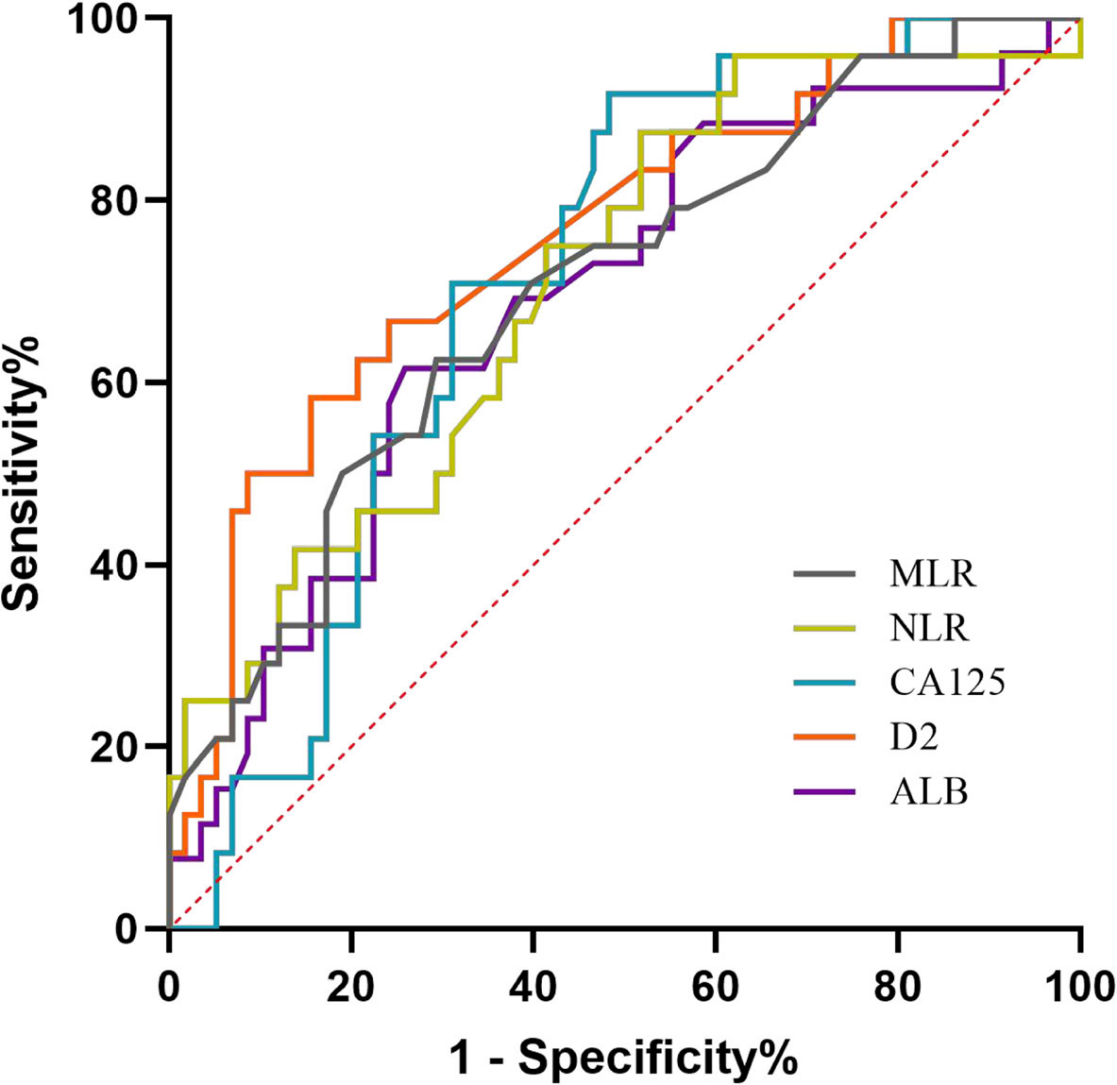
Receiver operating characteristic curve demonstrating the AUC of preoperative MLR, NLR, CA125, D2, and ALB for platinum resistance. (Abbreviations: AUC, the area under the curve; MLR, monocyte-to-lymphocyte ratio; NLR, neutrophil to lymphocyte ratio; CA125, carbohydrate antigen 125; D2, D-dimer; ALB, albumin)

ROC curves for platinum-based chemotherapy outcome prediction were generated to verify the optimal cut-off point for CA125, albumin, D-dimer, and NLR, MLR. The area under the curve (AUC) and the best cut-off values were established by plotting ROC curves (Table 2 and Fig. 1). The AUC of these curves ranged from the lowest value of 0.676 (albumin) to the highest value of 0.761 (D-dimer). The cut-off value, sensitivity, specificity of albumin to predict platinum resistance were ≤ 39.6 g/l, 58.3, and 74.1%, respectively. The corresponding D-dimer and NLR values were > 3.27 mg/l, 58.3, and 84.5% as well as > 2.28, 87.5, and 48.3%, respectively.

**Table 2:** Predictive values of preoperative blood parameters and NLR, MLR for determination of platinum resistance

Variables	AUC	*P*	95% CI	Cut-off value	Se%	Sp%	PPV%	NPV%
MLR	0.701	**0.004**	0.577–0.825	0.3	62.5	70.7	46.9	82.0
NLR	0.710	**0.003**	0.589–0.832	2.28	87.5	48.3	41.2	90.3
ALB	0.676	**0.013**	0.547–0.804	39.6	58.3	74.1	81.1	48.3
CA125	0.713	**0.003**	0.601–0.825	135.15	91.7	51.7	44.0	93.8
D2	0.761	**< 0.001**	0.646–0.876	3.27	58.3	84.5	60.9	83.1

*Abbreviations: MLR* monocyte to lymphocyte ratio, *NLR* neutrophil to lymphocyte ratio, *ALB* albumin, *CA125* carbohydrate antigen 125, *D2* D-dimer, *AUC* area under the curve, *CI* confidence interval, *Se* sensitivity, *Sp* specificity, *PPV* positive predictive value, *NPV* negative predictive value

*P* values with statistical significance were denoted

### Prognostic factors influencing long-term survival with platinum-based chemotherapy

**Fig. 2:**
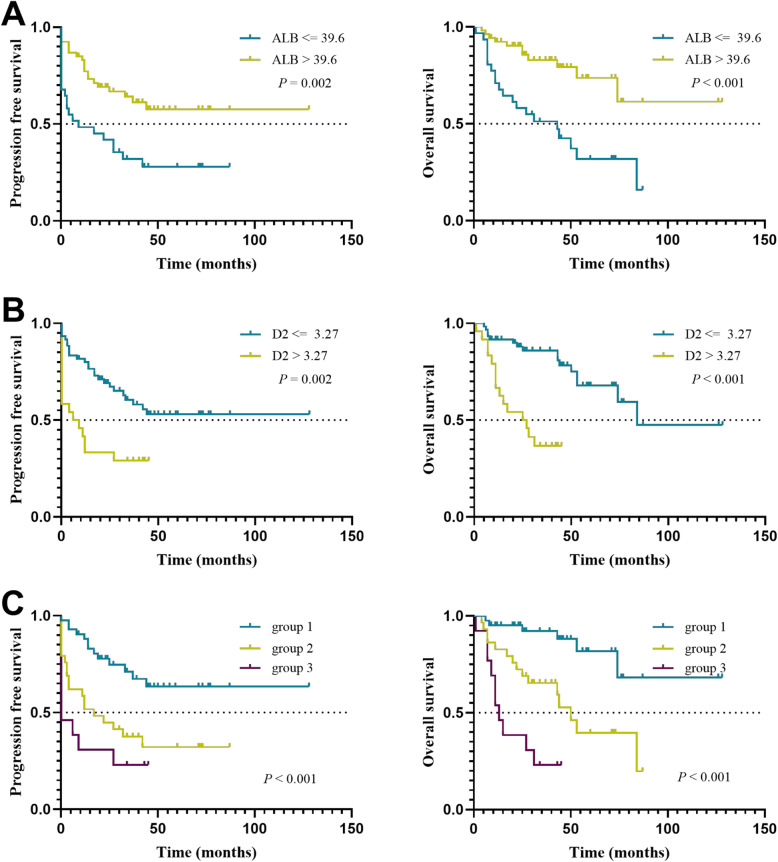
Kaplan-Meier curves showing PFS and OS stratified by preoperative ALB (**a**), D2 (**b**), and groups combining ALB and D2 (**c**). Group 1, high ALB/low D2; group 2, high ALB/high D2 and low ALB/low D2; group 3, low ALB/high D2. The *p*-values were calculated using the log-rank test. (Abbreviations: ALB, albumin; D2, D-dimer; PFS, progression-free survival; OS, overall survival)

We found that preoperative CA125, albumin, and D-dimer levels were significant prognostic indicators for OS and PFS. CA125 > 135.2 U/ml, albumin< 39.6 g/l, and D-dimer> 3.27 mg/l were associated with shorter PFS and OS (*P* < 0.05). In the univariate analysis, only NLR among the SIR markers had prognostic significance for PFS (*P* = 0.007). Multivariate analysis was performed on all these factors to eliminate the confounding effect. On multivariate analysis, patients with D-dimer > 3.27 mg/l (*P* = 0.001 for OS and *P* = 0.040 for PFS) and albumin < 39.6 g/l (*P* = 0.005 for OS and *P* = 0.041 for PFS) retained significance, respectively (Table 3 and Fig. 2a, b). CA125 was not highly correlated with OS (*P* = 0.074) and PFS (*P* = 0.054), and NLR (*P* = 0.103) was not related to PFS after adjusting for confounding variables.

**Table 3:** Univariate and multivariate cox proportional analysis regarding overall survival and progression free survival

Variables	OS						PFS					
	Univariate analysis			Multivariate analysis			Univariate analysis			Multivariate analysis		
	HR	95% CI	*P*	HR	95% CI	*P*	HR	95% CI	*P*	HR	95% CI	*P*
MLR ≤0.30 vs. > 0.30	/	/	0.061	/	/	0.882	/	/	0.079	/	/	0.943
NLR ≤2.28 vs. > 2.28	/	/	0.121	/	/	0.854	2.767	1.320–5.800	**0.007**	/	/	0.103
CA125 ≤ 135.2 vs. > 135.2	3.828	1.468–9.983	**0.006**	/	/	0.074	2.665	1.306–5.436	**0.007**	2.057	0.989–4.282	0.054
ALB ≤39.6 vs. > 39.6	0.279	0.134–0.584	**0.001**	0.345	0.163–0.731	**0.005**	0.404	0.220–0.743	**0.004**	0.521	0.279–0.973	**0.041**
D2 ≤ 3.27 vs. > 3.27	5.118	2.273–11.520	**< 0.001**	4.092	1.809–9.254	**0.001**	2.552	1.365–4.773	**0.003**	1.959	1.032–3.717	**0.040**

*Abbreviations: PFS* progression-free survival, *OS* overall survival, *MLR* monocyte to lymphocyte ratio, *NLR* neutrophil to lymphocyte ratio, *ALB* albumin, *CA125* carbohydrate antigen 125, *D2* D-dimer, *HR* hazard ratio, *CI* confidence interval

*P* values with statistical significance were denoted

**Fig. 3:**
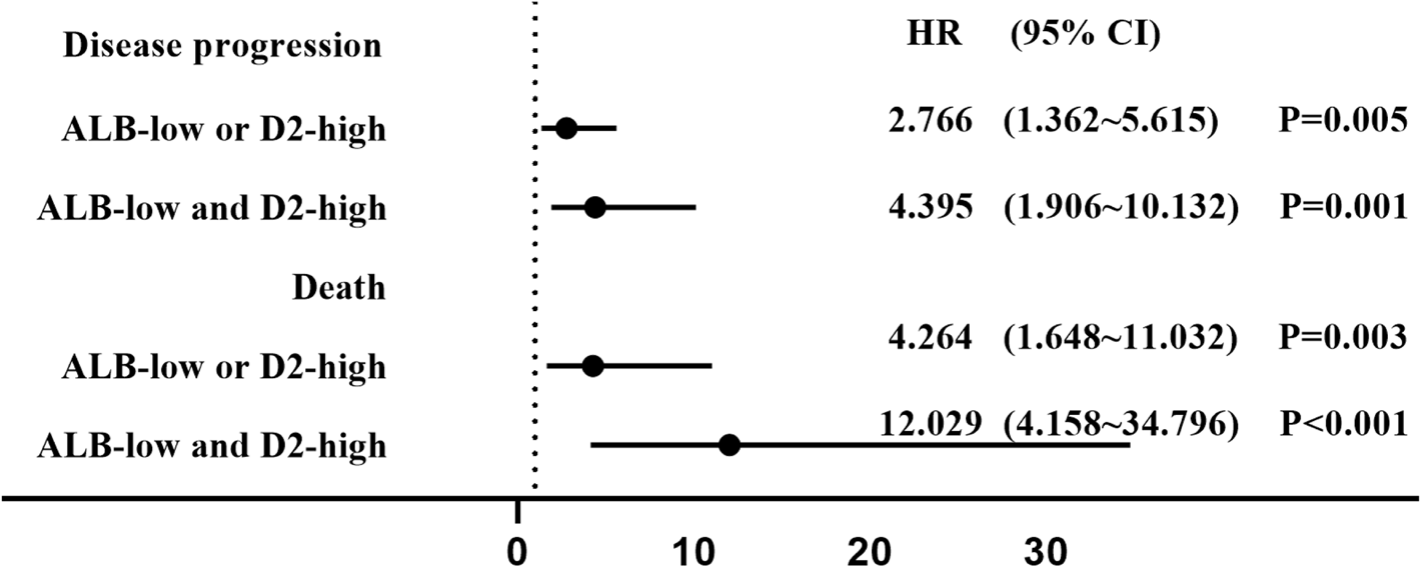
The risk of disease progression and death compared to ALB-high and D2-low. (Abbreviations: ALB, albumin; D2, D-dimer)

Then, we further stratified patients into three groups based on cut-off values of albumin (ALB) and D-dimer (D2). ALB lower than or D2 higher than the cut-off value was defined as abnormal. The grouping basis is as follows: group 1 = both normal (high ALB/low D2); group 2 = one abnormal (high ALB/high D2 and low ALB/low D2); group 3 = both abnormal (low ALB/high D2). The survival curves for the three groups are shown in Fig. 2c. Cox regression analysis showed that the risk of disease progression was 2.766-fold (95% CI, 1.362 ~ 5.615) and 4.395-fold {95% confidence interval (CI), 1.906 ~ 10.132} increased in groups 2 and 3 compared with group 1. The risk of death was 4.264-fold (95% CI, 1.648 ~ 11.032) and 12.029-fold (95% CI, 4.158 ~ 34.796) increased, respectively (Fig. 3).

## Discussion

Inflammation may play an important role in cancer progression. Increased neutrophils could promote tumor proliferation, angiogenesis, and invasion [14], whereas reduced lymphocyte levels indicate immune deficiency [15]. Therefore, the NLR reflects both inflammation and the immune system. A high pretreatment NLR is an adverse prognostic indicator for both the early and advanced stages of several malignancies [10, 16]. Emerging evidence showed that SIR markers, such as NLR, PLR, and MLR, were associated with the prognosis of ovarian cancer patients [17, 18]. To date, few reliable preoperative biomarkers that predict resistance or prognosis in OCCC have been identified. Four studies on the effectiveness of SIR markers in OCCC patients have been reported in the last 5 years, as shown in Table 4. Concerning NLR, most of the previous reports suggested that NLR was a prognostic indicator for PFS and/or OS [13, 19, 20]. There are some conflicting results regarding PLR. One study suggested that high PLR was associated with unfavorable outcomes, advanced stage, resistance to primary treatment, and decreased survival [19], whereas other studies did not. One of the studies found that the lymphocyte-to-monocyte ratio was an independent predictor of OS, whereas NLR and PLR were not [21].

**Table 4:** Summary of studies examining SIR as prognostic factors in OCCC patients

	Kim 2016 [19]	Zhang 2017 [20]	Kwon 2018 [12]	Yoshida 2019 [13]	The present study
Sample size	109	155	109	83	84
Advanced-stage %	37.5	29	41.3	0	44
platinum resistance %	18.3	12.9	22	NA	28.6
Prognostic factor for PFS	NLR, PLR	NLR	None	None	None
Prognostic factor for OS	None	NLR	LMR	NLR	None
Cut-off value	4.44 for resistance 2.8 for survival	NA	NA	3.26 for OS	2.28 for resistance

*Abbreviations: SIR* Systemic inflammatory response, *NLR* neutrophil to lymphocyte ratio, *PLR* platelet to lymphocyte ratio, *LMR* lymphocyte to monocyte ratio, PFS progression-free survival, OS overall survival, NA not available

In the present study, in addition SIR markers, we conducted a comprehensive analysis of blood cells and biochemical indicators, which might be related to OCCC patient survival. The most interesting finding was that despite a significant increase in preoperative platelets in OCCC patients, it was not associated with staging, availability of optimal surgery, or platinum resistance. Similarly, no correlation was noted between platinum resistance and preoperative lymphocyte count. Thus, PLR is not related to resistance or survival, which is consistent with most relevant studies [12, 13, 20]. Preoperative NLR level was associated with postoperative indicators, such as FIGO stage, residual tumor, and platinum resistance, which are known prognostic factors in OCCC [5]. The difference was that although univariate analysis showed NLR was significantly associated with PFS, the relation was not supported by multivariate analysis, indicating that NLR is not an independent predictor of survival. This discrepancy was potentially attributed to sample size differences and the different cut-off values used. In the present study, the proportion of patients with advanced-stage and platinum-resistant disease was the highest, and the NLR cut-off value was determined by whether patients were platinum sensitive or resistant. The original intention of the design is to address the major obstacle in the treatment of OCCC, which remains resistance to platinum-based chemotherapy. The finding suggests that a high NLR caused by an increased level of neutrophils reduce the response to adjuvant chemotherapy. However, its effect on PFS is influenced by additional factors.

Using stepwise comparison of prognostic values among the potential markers, we sought to identify the most dominant markers related to chemotherapy resistance and clinical outcomes in IC-IV OCCC. We found that preoperative D-dimer and albumin levels in OCCC patients were significantly correlated with platinum resistance and were independent predictors of PFS and OS. Patients with cancer often exhibit a state of hypercoagulation and exaggerated fibrinolysis [22]. D-dimer, as an end product of fibrinogen, is a signal of the activated coagulation system in numerous cancer types especially in the advanced stage [23, 24]. Emerging studies also suggest that a high pretreatment plasma D-dimer levels are a poor prognostic factor in EOC [8, 25]. Regarding OCCC patients, in whom D-dimer levels are generally elevated and are more pronounced compared with other EOC patients, much attention has been paid to the relationship between D-dimer and venous thromboembolism [7, 26]. The present study reported that a D-dimer cut-off value of 3.27 is a useful predictor of chemoresistance and can be used as an independent predictor of PFS and OS in clear cell ovarian cancer patients. On the other hand, pretreatment hypoalbuminemia, which is the outcome of malnutrition and cachexia in cancer patients due to the host responses to the tumor, also provides prognostic significance in OCCC [20]. Consistent with previous studies, we found out that hypoalbuminemia (albumin cut-off point of 39.6) and D-dimers acted as independent predictors of PFS and OS. Moreover, the risk of disease progression and death significantly increased if either the albumin or D-dimer cut-off value was surpassed. The corresponding risks of patients who reached albumin and D-dimer cut-off values were 4-fold and 12-fold increased, respectively, compared with those who did not. Thus, D-dimer and albumin may play an important role in selecting patients for adjuvant anti-cancer therapy. For OCCC patients with a high possibility of platinum resistance, high recurrence rate, and mortality, it is worth discussing whether early intervention using other anti-tumor therapies, such as targeted drugs, should be considered.

Pretreatment plasma D-dimer and albumin levels were each identified as prognostic factors for several malignancies [23, 24, 27], including ovarian cancer [9, 25]; however, few studies have combined them to assess their role in chemoresistance. Given that OCCC exhibits increased D-dimer expression compared with other ovarian cancers, this combined evaluation seems to be more valuable in OCCC patients. Nevertheless, their AUCs were not greater than 0.8, indicating that these findings could not be directly applied to clinical practice. Current studies that have reported that HE4 and mesothelin could be used as ovarian tumor markers [28–31]. Additionally, one study showed that HE4 protein promoted the proliferation of ovarian cancer cells and resistance to carboplatin in vitro, suggesting the value of HE4 in predicting the growth potential of epithelial ovarian cancer tumors and platinum resistance [32]. It is hoped that more potential biomarkers can be used and combined to improve the sensitivity and specificity of the evaluation. Further validation of these easily available parameters as promising prognostic biomarkers for patients with OCCC in prospective studies is encouraged.

The present study presents several limitations. First, selection and surveillance biases in our analysis could not be controlled due to the retrospective study design of only 84 samples from a single academic institution. Second, although we excluded patients with any inflammatory condition, some hematological biomarkers may have been affected by the presence of unrecognized systemic inflammatory diseases. Third, some possible confounders affecting SIR markers and coagulation function were not assessed.

## Conclusion

Elevated levels of preoperative D-dimer and low albumin levels may be the most useful biomarkers of worse response to first-line platinum-based chemotherapy and poor clinical outcomes. Elevated NLR has some predictive value for platinum resistance, but its predictive effect on prognosis requires further large-scale prospective investigation.

## Data Availability

The dataset supporting the conclusions of this article is available upon request. Please contact Dr. Shuang Ye (mendy_ye@126.com).

## Abbreviations

EOC: epithelial ovarian cancer
OCCC: ovarian clear cell carcinoma
FIGO: the International Federation of Gynecology and Obstetrics
SIR: systemic inflammatory response
NLR: neutrophil-to-lymphocyte ratio
MLR: monocyte-to-lymphocyte ratio
CA125: carbohydrate antigen 125
PLR: platelets to lymphocyte ratio
ROC: receiver operating characteristic
PFS: progression-free survival
OS: overall survival
AUC: area under the curve
CI: confidence interval
